# Distinct Fcα receptor *N*-glycans modulate the binding affinity to immunoglobulin A (IgA) antibodies

**DOI:** 10.1074/jbc.RA119.009954

**Published:** 2019-07-30

**Authors:** Kathrin Göritzer, Aysegül Turupcu, Daniel Maresch, Jan Novak, Friedrich Altmann, Chris Oostenbrink, Christian Obinger, Richard Strasser

**Affiliations:** ‡ Department of Applied Genetics and Cell Biology, University of Natural Resources and Life Sciences, Muthgasse 18, A-1190 Vienna, Austria; § Department of Material Sciences and Process Engineering, University of Natural Resources and Life Sciences, Muthgasse 18, A-1190 Vienna, Austria; ¶ Department of Chemistry, Division of Biochemistry, University of Natural Resources and Life Sciences, Muthgasse 18, A-1190 Vienna, Austria; ‖ Department of Microbiology, University of Alabama, Birmingham, Alabama 35294

**Keywords:** glycobiology, glycosylation, glycoprotein structure, immunoglobulin A (IgA), Fc receptor, molecular modeling, antibody, recombinant protein expression, adaptive immunity, posttranslational modifications

## Abstract

Human immunoglobulin A (IgA) is the most prevalent antibody class at mucosal sites with an important role in mucosal defense. Little is known about the impact of *N*-glycan modifications of IgA1 and IgA2 on binding to the Fcα receptor (FcαRI), which is also heavily glycosylated at its extracellular domain. Here, we transiently expressed human epidermal growth factor receptor 2 (HER2)-binding monomeric IgA1, IgA2m(1), and IgA2m(2) variants in *Nicotiana benthamiana* ΔXT/FT plants lacking the enzymes responsible for generating nonhuman *N*-glycan structures. By coinfiltrating IgA with the respective glycan-modifying enzymes, we generated IgA carrying distinct homogenous *N*-glycans. We demonstrate that distinctly different *N*-glycan profiles did not influence antigen binding or the overall structure and integrity of the IgA antibodies but did affect their thermal stability. Using size-exclusion chromatography, differential scanning and isothermal titration calorimetry, surface plasmon resonance spectroscopy, and molecular modeling, we probed distinct IgA1 and IgA2 glycoforms for binding to four different FcαRI glycoforms and investigated the thermodynamics and kinetics of complex formation. Our results suggest that different *N*-glycans on the receptor significantly contribute to binding affinities for its cognate ligand. We also noted that full-length IgA and FcαRI form a mixture of 1:1 and 1:2 complexes tending toward a 1:1 stoichiometry due to different IgA tailpiece conformations that make it less likely that both binding sites are simultaneously occupied. In conclusion, *N*-glycans of human IgA do not affect its structure and integrity but its thermal stability, and FcαRI *N*-glycans significantly modulate binding affinity to IgA.

## Introduction

Glycosylation is an important co- and posttranslational modification that affects many properties of proteins, including folding, stability, subcellular localization, and interaction with other proteins. A very prominent example for the important role of glycosylation is the single *N*-glycan in the CH2 domain of the Fc region of immunoglobulin (Ig) G. The presence of this *N*-glycan is crucial to maintain an open conformation of the Fc domain (1) and influences properties like conformational and thermal stabilities (2). Furthermore, it was found that specific *N*-glycan modifications such as removal of the core fucosylation lead to increased binding affinity to the Fcγ receptor IIIa (3–6) due to increased carbohydrate–carbohydrate interactions with the *N*-glycans of the Fcγ receptor promoting substantially increased antibody-dependent cellular cytotoxicity (7).

Although the impact of IgG Fc *N*-glycans on Fcγ receptor binding is well-studied, the role of *N*-glycans on recombinant and endogenous human receptor has been characterized only recently (8, 9). These studies revealed cell type–dependent differences in *N*-glycan composition and showed, for example, that oligomannosidic *N*-glycans lead to a 12-fold increase in affinity of the Fcγ receptor IIIa to IgG1 Fc (10). These data suggest that both the IgG Fc *N*-glycan modifications and the presence of distinct *N*-glycans on the corresponding receptor contribute to the modulation of the immune response.

Surprisingly, despite the great importance of IgG glycosylation, little is known about the role of glycans for other Ig isotypes. Human IgA, the predominant antibody at mucosal sites, occurs in two subclasses, IgA1 and IgA2, and for IgA2 there are two major allotypes (IgA2m(1) and IgA2m(2)). All these IgA variants are extensively glycosylated. IgA1 has two *N*-glycans, one in the CH2 domain and one in the tailpiece and several *O*-glycosylation sites in the extended hinge region. IgA2 variants lack *O*-glycans but carry four to five *N*-glycans on the heavy chain (11).

Instead of stabilizing intramolecular interactions between the two heavy chains of IgG, the *N*-glycan in the IgA1 CH2 domain is located at the surface of the protein and may influence the conformation of the protein and its binding to different receptors (12). Available data about the interaction of IgA with the FcαRI^2^ do not give a clear answer whether distinct IgA glycans play a role in the receptor interaction (13–17). The use of IgA isolated from human serum or recombinant IgA produced in mammalian cells bearing rather heterogeneous *N*-glycans has complicated the interpretation of the results. This also applies to FcαRI, which is heavily glycosylated with six predicted *N*-glycosylation and nine putative *O*-glycosylation sites at its extracellular domain.

Here, we used a plant-based glycoengineering approach to generate IgA1, IgA2m(1), and IgA2m(2) carrying distinct homogeneous *N*-glycans. Additionally, we produced four distinct glycoforms of the extracellular domain of FcαRI in HEK293F cells. This approach allowed a detailed investigation of the impact of the *N*-glycosylation of the two IgA subclasses and of the extracellular domain of FcαRI on the thermodynamics and kinetics of complex formation by using a broad array of biochemical and biophysical methods, including size-exclusion chromatography coupled to multiangle light scattering (SE-HPLC-MALS), differential scanning calorimetry (DSC), isothermal titration calorimetry (ITC), and surface plasmon resonance (SPR) spectroscopy as well as molecular modeling and simulation.

## Results

### Production of IgA isotypes with defined N-glycans

**Figure 1.**
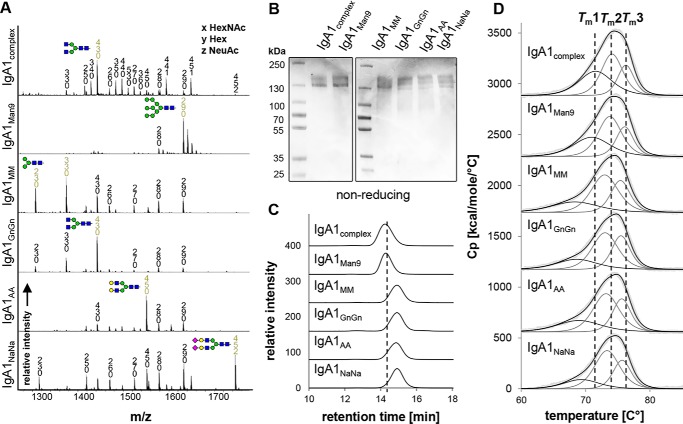
***N*-Glycan composition and thermal stability of IgA1 glycoforms.** *A*, representative MS spectra ([M + 3H]^3+^) of the tryptic glycopeptide LSLHRPALEDLLLGSEANLTCTLTGLR containing the CH2-resident NLT glycosylation site derived from the α chain of the different purified IgA1 glycoforms are shown. IgA1_complex_ and IgA1_Man9_ are HEK293F-derived; all other variants are plant-produced. *N*-Glycans are abbreviated according to the ProGlycAn system (www.proglycan.com).^3^ The *symbols* for the monosaccharides are drawn according to the nomenclature from the Consortium for Functional Glycomics (http://www.functionalglycomics.org/).^3^ Illustrations of selected major peaks are shown. *B*, SDS-PAGE of purified IgA1 glycoforms under nonreducing conditions followed by Coomassie Brilliant Blue staining. *C*, SE-HPLC measurements of the different IgA1 glycoforms. To facilitate comparison between the different variants, the elution time of IgA1_complex_ is marked with a *dashed line. D*, DSC analysis of the IgA1 glycoforms. The *black lines* show fitted representative thermograms, whereas the *gray lines* are the deconvoluted peaks of each domain transition, and the *light gray lines* are the raw data. For comparison, the three midterm transitions of the CH2, Fab, and CH3 domains of IgA1_complex_ produced in HEK293F cells are marked with *dashed lines. Cp*, heat capacity.

To assess the impact of *N*-glycosylation on structure–function relationships, different IgA variants bearing homogenous *N*-glycans were generated. IgA1 produced in HEK293F cells has very heterogeneous complex *N*-glycans with high amounts of branched and incompletely sialylated structures (18) (Fig. 1*A*, IgA1_complex_). A more homogenous *N*-glycosylation profile was obtained by expressing IgA1 in HEK293F cells in the presence of the class I α-mannosidase inhibitor kifunensine, resulting in IgA1 with exclusively oligomannosidic *N*-glycans (Fig. 1*A*, IgA1_Man9_). To produce additional glycoforms, we expressed IgA1 transiently in glycoengineered *Nicotiana benthamiana*, which are capable of producing glycoproteins with homogenous human-like *N*-glycans (19, 20). IgA1 carrying mostly biantennary GlcNAc_2_Man_3_GlcNAc_2_ (GnGn) complex *N*-glycans was produced in the ΔXT/FT line (21) by overexpressing a *trans*-Golgi–targeted human *N*-acetylglucosaminyltransferase II (GnTII). Coexpression of two different *N. benthamiana* β-hexosaminidases targeted to the *trans*-Golgi and to the apoplast resulted in the formation of Man_3_GlcNAc_2_ (MM) and GlcNAc_1_Man_3_GlcNAc_2_ *N*-glycan structures (Fig. 1*A*, IgA1_MM_). IgA1 with predominately terminally galactosylated glycans (Fig. 1*A*, IgA1_AA_) was obtained by coexpression of GnTII and β1,4-galactosyltransferase (22). Finally, IgA1 with terminally sialylated *N*-glycans (Fig. 1*A*, IgA1_NaNa_) was produced by further coexpressing the entire CMP-*N*-acetylneuraminic acid (CMP-Neu5Ac) biosynthesis pathway, the transporter that delivers CMP-sialic acid to the Golgi, and the α2,6-sialyltransferase to transfer CMP-sialic acid to terminal galactose on the glycoprotein (20).

The transient coexpression of IgA2m(1) and IgA2m(2) isotypes with the respective *N*-glycan–processing enzymes enabled the modification of their oligosaccharides in a similar manner as described for IgA1 (Fig. S1). However, the efficiency of *N*-glycan modifications varied between different *N*-glycosylation sites and IgA isotypes. Although the CH2-resident site (NLT) could be modified very efficiently in IgA1 (Fig. 1*A*), the extension to terminally sialylated *N*-glycans was less efficient on the corresponding site in the IgA2 isotypes (Fig. S1). In the latter, this *N*-glycosylation site is in close proximity to another CH2-resident site (NIT), which displays high amounts of sialylated (NaNa) *N*-glycans. Furthermore, also the underglycosylated NVS site in the tailpiece of IgA1 and IgA2 isotypes displayed reduced amounts of modified *N*-glycans compared with other IgA *N*-glycosylation sites (18, 23). Taken together, using expression in HEK293F cells and in glycoengineered plants, the production of IgA variants with tailored *N*-glycans (glycoforms) was achieved.

### Characterization of different IgA glycoforms

The purified monomeric IgA glycoforms were investigated for their overall assembly and homogeneity using SDS-PAGE and SE-HPLC coupled to MALS. The nonreducing SDS-PAGE of the purified IgA1 glycoforms shows a predominant band at a molar mass around 160 kDa for each variant (Fig. 1*B*), representing the fully assembled antibody without the presence of any degradation products or impurities. The size-exclusion chromatography (SEC) profiles gave narrow single and monodisperse peaks. The retention time shifts due to different *N*-glycan composition (Fig. 1*C*). HEK293F-produced IgA1_complex_ and IgA1_Man9_ displayed the shortest retention time followed by the plant-produced IgA1_NaNa_, IgA1_AA_, IgA1_GnGn_, and IgA1_MM_ glycoforms. This hierarchy also applied to the IgA2m(1) and IgA2m(2) isotypes (Fig. S2). The shift toward longer retention times in plant-produced IgA variants compared with HEK-derived IgAs results from their lower molecular masses confirmed by MALS due to a higher degree of underglycosylation of the tailpiece *N*-glycosylation site (Fig. S3) (18, 23).

Next, the thermal stability of the IgA variants was investigated by DSC. As described previously, the thermal unfolding of IgA is represented by a broad endotherm, which suggests the presence of three independent transitions (1). This allows the identification of the transition midpoint temperatures of the CH2 (*T_m_*1), Fab (*T_m_*2), and CH3 (*T_m_*3) domains as already described for IgG (18, 24). IgA1_complex_ produced in HEK293F cells exhibited melting temperatures at 71.5 ± 0.06 (T*_m_*1), 74.2 ± 0.04 (T*_m_*2), and 76.3 ± 0.05 °C (T*_m_*3) (Fig. 1*D*), respectively, that are in accordance with previously reported endotherms of IgA1 produced in HEK293-6E cells that were measured on a different DSC machine (18). Although the HEK293F-produced IgA1_Man9_ showed slightly decreased thermal unfolding temperatures of the CH2 domain, this effect was more pronounced in plant-derived variants. However, the stability of the CH2 domain of plant-produced IgA1 slightly increased with more extended *N*-glycans with the hierarchy of thermal stability being IgA1_NaNa_ > IgA1_AA_ > IgA1_GnGn_ > IgA1_MM_. This correlation has been observed for the IgA2m(1) and IgA2m(2) subclasses as well (Table S1).

Overall, our findings indicate an effect of *N*-glycan modifications on thermal stability. However, the difference in thermal stability of HEK293F- and plant-derived IgA1 and IgA2 variants could additionally be attributed to a higher degree of underglycosylation of the tailpiece *N*-glycosylation site of plant-produced IgA. Furthermore, the different *O*-glycan modifications in the hinge region of plant- and HEK293F-derived IgA1 might contribute to thermal stability as well. IgA1 has nine potential *O*-glycosylation sites in its extended proline-rich hinge region of which six are found to be occupied with a combination of mucin-type core structures in HEK293F-derived IgA1. On the hinge region of plant-produced IgA1, in contrast, conversion of proline residues to hydroxyproline and the presence of additional pentoses are observed that might destabilize plant-derived IgA1 (18, 25, 26). Together with *N*-glycan modifications, these different hinge region *O*-glycans could also contribute to the observed differences in thermal unfolding of the CH2 domain.

Finally, binding to the antigen HER2 was assessed by ELISA, and the EC_50_ was determined for the different IgA1 glycoforms. As expected, antigen binding behavior of all IgA1 glycoforms is very similar and independent of glycosylation or production host (Fig. S4).

### Analysis of FcαRI glycosylation

**Figure 2.**
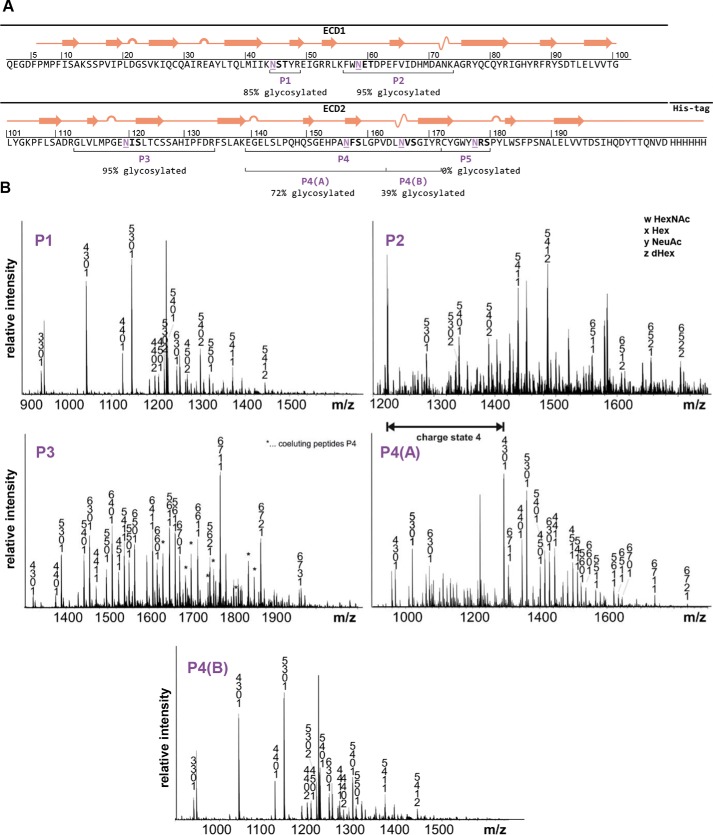
***N*-Glycan characteristics of the recombinant extracellular domain of FcαRI.** *A*, schematic representation of the secondary structure of the HEK293F-produced extracellular domain (*ECD*). Putative *N*-glycosylation sites are marked in *purple* and are *underlined*. The degree of *N*-glycan site occupancy (percentage of glycosylation) is indicated for each site, and the obtained peptides are highlighted and marked P1–P5. *B*, representative MS spectra ([M + 3H]^3+^) of the glycopeptides P1–P4(B) obtained from digested recombinant FcαRI.

Previous reports indicated the contribution of distinct Fcγ receptor *N*-glycans to IgG binding (10). It is possible that the *N*-glycan composition of FcαRI plays a similar role for IgA1 interaction. In the crystal structure of the IgA1-Fc in complex with a FcαRI produced in insect cells, distinct *N*-glycans of the receptor are in close proximity to the interaction surface and are suspected to influence receptor binding (12). Compared with humans, insect cell *N*-glycans are significantly smaller, and thus the receptor with human-type complex *N*-glycans might be even closer to the interaction surface than it appears in the crystal structure (27). Although a role of FcαRI *N*-glycosylation for IgA binding has been described (28), no site-specific information about the *N*-glycan composition of FcαRI sites is available yet. There are six predicted *N*-glycosylation and nine putative *O*-glycosylation sites in the extracellular domain of human FcαRI (CD89). To assess the *N*-glycosylation status of recombinant FcαRI, the extracellular domain was expressed in HEK293F cells. Purified FcαRI was digested with trypsin as well as Asp-N and analyzed by LC-electrospray ionization-MS to determine the *N*-glycosylation status and site-specific *N*-glycan composition (Fig. 2*A*). Not all predicted *N*-glycosylation sites were found to be occupied. Although the first three N-terminal glycosylation sites Asn-44, Asn-58, and Asn-120 are fully occupied, the sites Asn-56 and Asn-165 on tryptic peptide 4 are incompletely glycosylated, and the C-terminal *N*-glycosylation site Asn-177 was only found unglycosylated. The *N*-glycans found on the recombinant receptor are very heterogenous and display site-specific variations in terms of level of branching, galactosylation, and sialylation (Fig. 2*B*). Generally, the peaks correspond to complex-type biantennary or branched *N*-glycans with high levels of fucosylation as well as incomplete galactosylation and sialylation. Several of the detected glycopeptide masses could not be assigned to a distinct glycan composition because of the possible presence of multiple isobaric structures, including different branched complex *N*-glycans with or without a bisecting *N*-acetylglucosamine (GlcNAc) and modification on the nonreducing end. MS2 spectra of the N-terminal glycopeptide indicate the presence of fucosylation on the nonreducing end rather than core fucosylation. Furthermore, investigation of the tryptic peptides of a recombinant FcαRI that only has single GlcNAc residues at each *N*-glycosylation site (see next section) did not indicate the presence of any additional *O*-glycan modifications.

### Production and characterization of different FcαRI glycoforms

**Figure 3.**
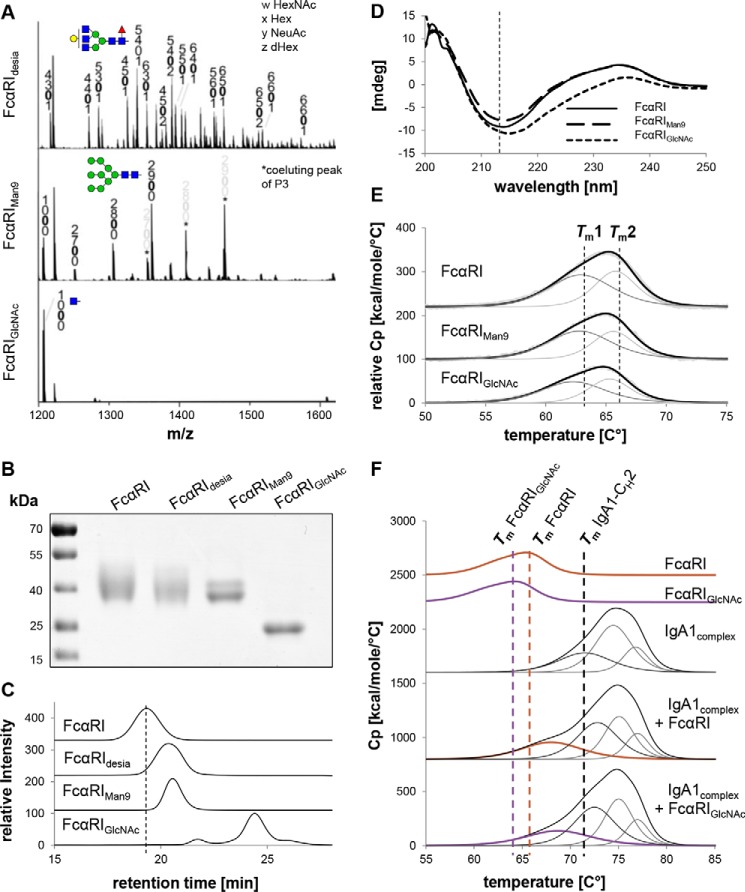
**Homogeneity and thermal stability of different FcαRI glycoforms.** *A*, representative MS spectra of tryptic glycopeptide P2 obtained from digested FcαRI_desia_ ([M + 3H]^3+^), FcαRI_Man9_ ([M + 3H]^3+^), and FcαRI_GlcNAc_ ([M + 3H]^2+^). *B*, SDS-PAGE of the different purified FcαRI glycoforms under reducing conditions. Proteins were detected by Coomassie Brilliant Blue staining. *C*, SE-HPLC measurements of the FcαRI glycoforms. To facilitate comparison between the different variants, the elution time of the FcαRI with complex sialylated *N*-glycosylation is marked with *dashed lines. D*, CD analysis of different FcαRI glycoforms. The CD spectrum minimum of FcαRI at 214 nm is marked in *dashed lines* for comparison. *E*, DSC analysis of different FcαRI glycoforms. The *black lines* show fitted representative DSC thermograms, whereas the *gray lines* are the deconvoluted peaks of each domain transition, and the *light gray lines* are the raw data. For comparison, the two midterm transitions of each FcαRI domain are marked with *dashed lines. F*, DSC analysis of different FcαRI glycoforms and IgA1–FcαRI complexes mixed in a molar ratio of 1:1. *Bold lines* show fitted representative DSC thermograms, whereas the *thin lines* are the deconvoluted peaks of each domain transition. For comparison, the midterm transitions of FcαRI, FcαRI_GlcNAc_, and the CH2 domain of IgA1 are marked with *dashed lines. mdeg*, millidegrees. *Cp*, heat capacity.

Next, the influence of different *N*-glycan modifications on the structure and function of FcαRI was investigated. Therefore, receptor variants with either complex sialylated (FcαRI), complex desialylated (FcαRI_desia_), or oligomannosidic (FcαRI_Man9_) *N*-glycans as well as a variant with single GlcNAc residues attached to Asn (FcαRI_GlcNAc_) were generated (Fig. 3*A*) and subjected to thorough biochemical and biophysical investigation. Upon SDS-PAGE, 40-kDa bands of the purified receptor variants were detected except for the variant with single GlcNAc residues, which appeared as 25-kDa band (Fig. 3*B*). SE-HPLC runs gave single monodisperse peaks for all glycosylated variants with molecular masses of 42 kDa for the FcαRI, 40 kDa for FcαRI_desia_, and 37 kDa for FcαRI_Man9_, confirmed by MALS (Figs. 3*C* and S3). The FcαRI_GlcNAc_ variant having a molecular mass of 25.5 kDa displays two additional small peaks, suggesting disturbed conformational integrity.

To further investigate the effect of different *N*-glycan modifications on the overall conformation of the receptor, CD spectroscopy experiments were carried out. Therefore, far-UV spectra between 260 and 200 nm were recorded for the FcαRI, FcαRI_Man9_, and FcαRI_GlcNAc_ variants (Fig. 3*D*). The CD spectrum of the FcαRI exhibits a minimum at 214 nm, representing the predominant presence of β-sheets and low abundance of α-helical structures, which is in accordance to the crystal structure of FcαRI (12). The spectrum of FcαRI_Man9_ is very similar, whereas FcαRI_GlcNAc_ displays a shift of the minima, suggesting an alteration in the secondary structure. It has to be noted that also the lack of sugar moieties in the FcαRI_GlcNAc_ can cause a slight shift in absorbance because carbohydrates themselves also represent a small CD signal (29).

Next, the influence of this alteration in the secondary structure on the thermal stability of the FcαRI_GlcNAc_ variant was analyzed using differential scanning calorimetry. All FcαRI glycoforms showed nearly identical endotherms with FcαRI_GlcNAc_ being slightly destabilized by ∼1.5 °C (Fig. 3*E*). Furthermore, rescans revealed the capability of FcαRI to refold, whereas this feature is lost in the variant with single GlcNAc residues (Fig. S5).

### The impact of IgA and FcαRI glycans on the kinetics and thermodynamics of complex formation

**Figure 4.**
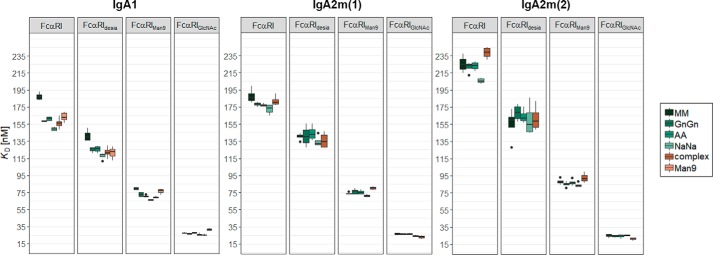
**The effect of *N*-glycans on binding affinities of IgA1, IgA2m(1), and IgA2m(2) to FcαRI.** *K_D_* values were obtained by SPR spectroscopy in single-cycle kinetic experiments from four independent measurements of two different receptor preparations. IgA1 glycoforms were captured on a Protein L chip, and increasing concentrations of the respective FcαRI glycoforms were injected. The obtained curves were fitted with a 1:1 binding model. *Error bars* represent S.D.

To examine the role of the *N*-glycans for the IgA and FcαRI binding, SPR spectroscopy with different IgA and FcαRI glycoforms was conducted. Thereby the different IgA1, IgA2m(1), and IgA2m(2) glycoforms were immobilized on a Protein L chip in an oriented manner with the Fc domain pointing toward the solution. Next, single-cycle kinetic experiments were carried out by injecting five increasing concentrations of the different FcαRI variants. Although the crystal structure of the IgA1–FcαRI complex suggests a 1:2 stoichiometry (12, 30), the obtained response units of the binding curves in SPR experiments proposed a 1:1 binding model, and the sensorgram was fitted accordingly. Using this setup, medium binding affinities to the FcαRI with *K_D_* values between 150 and 250 nm were obtained for HEK293F- and plant-produced IgA with a general hierarchy of affinity of IgA1 > IgA2m(1) > IgA2m(2) (Fig. 4). Characteristic for the interaction of all IgA–FcαRI complexes was a rapid association and dissociation, which have also been described in previous studies (30–32). IgA2 isotypes showed decreased association and dissociation rates compared with IgA1. Moreover, the differences in binding affinity observed for all different IgA and FcαRI glycoforms were mediated by the association rate (Table S2). The influence of the IgA *N*-glycans on the binding affinity was reproducible but small with the sialylated glycoform IgA1_NaNa_ being the best binder, an effect that cannot be observed to the same extent in the IgA2 isotypes (Fig. 4). This could be due to the less complete modification of the NVS-site glycans in these variants or a different conformational orientation of the IgA2 glycans.

The IgA1 *O*-glycosylation sites in the hinge regions are located 20–30 Å away from the FcαRI–IgA1-interacting region. They are therefore not expected to interact with the receptor and would at most contribute by minor long-range electrostatic effects or by causing conformational changes of IgA1 (33). Moreover, *O*-glycans are different in plant- and HEK293F-produced IgA1 (18), but the measured affinities toward FcαRI of plant- and HEK293F-derived IgA1 are comparable.

In contrast to the IgA *N*-glycans, FcαRI *N*-glycans contributed significantly to the binding interaction (Fig. 4). Although a slight increase of binding affinity of the desialylated FcαRI_desia_ could be observed, modification of the *N*-glycans toward oligomannosidic structures as well as single GlcNAc residues had more dramatic effects. The FcαRI_Man9_ variant was able to bind all glycoforms of the three different IgA isotypes 2 to 3 times better than the variants with complex *N*-glycans. The single GlcNAc-containing variant even displayed a 10-fold increase in binding affinity. This correlation is also found in all glycoforms of the IgA2 isotypes (Fig. 4) and is consistent with a previous study (28).

Next, to investigate whether the instability due to the removal of most of the *N*-glycans can be compensated through stabilization of the complex, DSC of FcαRI or FcαRI_GlcNAc_ in a 1:1 complex with IgA1 was carried out. In complex with IgA1, the FcαRI undergoes a significant increase in thermal stability that is even more pronounced in the IgA1–FcαRI_GlcNAc_ complex. Furthermore, also the CH2 domain of IgA1 shows an increase of the transition midpoint temperature from 71.5 to 72.8 °C in both complexes (Fig. 3*F*).

### Stoichiometry of the IgA–FcαRI interaction

**Figure 5.**
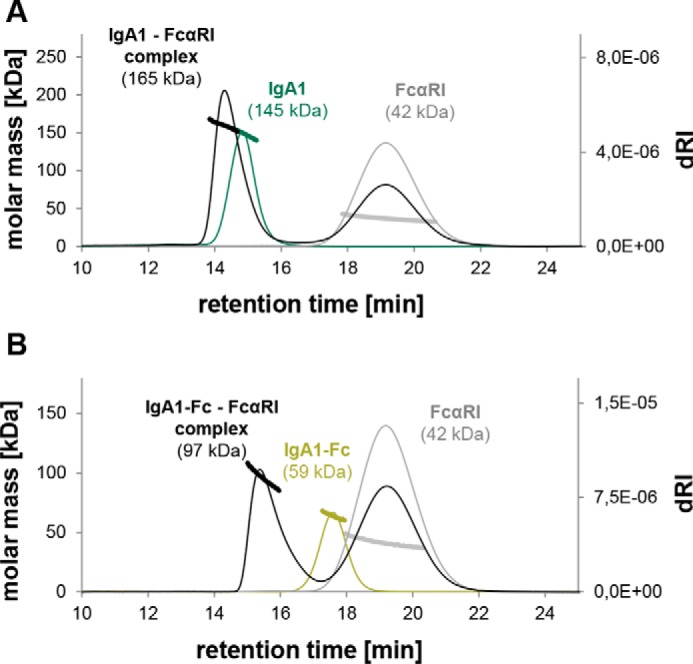
**SE-HPLC-MALS reveals molar masses of IgA1 and FcαRI complexes that correspond to a 1:1 stoichiometry.** *A*, overlay of the elution profile of IgA1 (*green*), FcαRI (*gray*), and a mixture of IgA1 and FcαRI in a ratio of 1:4 (*black*). *B*, overlay of the elution profile of IgA1-Fc (*light green*), FcαRI (*gray*), and a mixture of IgA1-Fc and FcαRI in a ratio of 1:4 (*black*). Note that the depicted molar masses are derived from MALS measurements and thus do not exactly match the exact molar masses. *dRI*, differential refractive index.

Unexpectedly, SPR experiments suggested a 1:1 binding model of the IgA–FcαRI complex, which is in discrepancy to the suggested 1:2 stoichiometry found in the crystal structure and ultracentrifugation experiments in previous studies (12, 30, 34). To investigate this further, mixtures of the different IgA variants with the various FcαRI variants were applied to SE-HPLC coupled to MALS to determine the molecular mass of the complex. IgA1_GnGn_ and FcαRI applied in a molar ratio of 1:4 formed a complex at around 165 kDa (Fig. 5*A*), which is below the theoretical mass of a 1:1 complex (187 kDa). Similar masses close to 1:1 complexes were revealed for all other IgA–FcαRI combinations (Fig. S6). Furthermore, an HEK293F-produced IgA1-Fc deletion protein lacking the Fab domain was analyzed in the same manner to exclude the possibility that the Fab arms interfere with the complex formation. The SEC profiles of the mixture of IgA1-Fc and FcαRI showed the formation of a stable complex at 97 kDa that corresponds closely to the theoretical mass of a 1:1 complex (105 kDa) (Fig. 5*B*).

**Figure 6.**
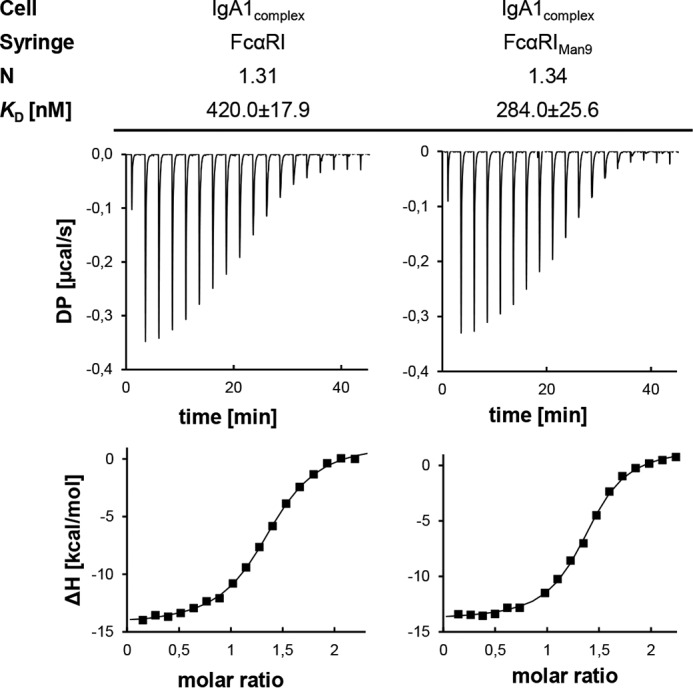
**Isothermal titration calorimetry indicates the same affinity for the first and second binding events of IgA1 to FcαRI.** The *upper panels* show the raw data representing the response to 19 injections at 25 °C, and the *lower panels* show the integrated data. *DP*, differential power.

The discrepancy in binding stoichiometry between the crystal structure (1:2) and our SPR and SEC analyses (1:1) could be caused by a cooperative binding behavior in which the binding of free IgA1 to FcαRI might show a significant higher affinity than the interaction of the IgA1–FcαRI complex with the second FcαRI (35). To investigate this hypothesis, ITC was performed. IgA protein samples were used at high concentrations in a sample cell to which highly concentrated receptor was titrated. In this setup, the ligand cannot be separated spatially from its receptor after dissociation, which, however, is possible during SEC. ITC binding experiments of IgA1_complex_ and IgA2m(2)_complex_ with either FcαRI or FcαRI_Man9_ exhibited a single transition, giving a 1:1.3 binding stoichiometry for each IgA–receptor combination (Figs. 6 and S7). This suggests the same affinity of the first and second binding events of FcαRI, whereas only 30% of the time a 1:2 complex was present. The obtained binding affinities of IgA1 to FcαRI and FcαRI_Man9_ were approximately 2.5-fold lower compared with the conducted SPR experiments but similar for IgA2m(2) binding to FcαRI and FcαRI_Man9_. This difference could be attributed to the IgA immobilization on a surface in SPR experiments, whereas ligand and analyte are in solution during ITC (Fig. S7).

### Molecular modeling of the IgA1 and FcαRI interaction

**Figure 7.**
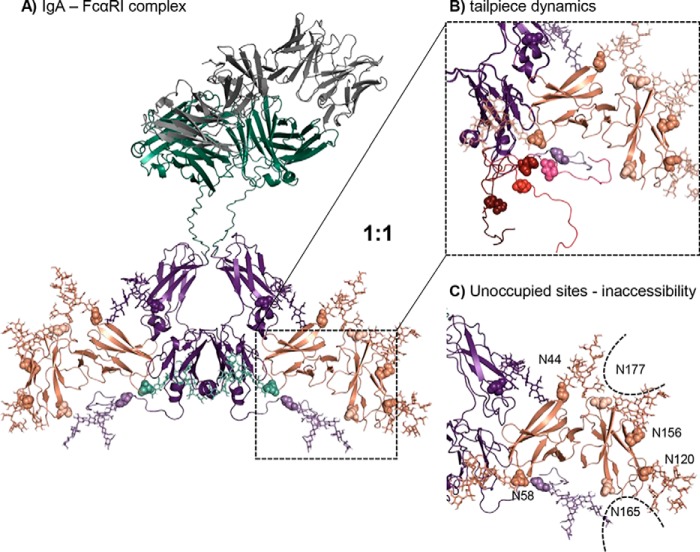
**The molecular model of *N*-glycosylated IgA1-Fc in complex with FcαRI suggests a 1:1 binding stoichiometry.** *A*, IgA1-Fc region colored in *purple*, constant heavy and light chains colored in *green*, variable region colored in *gray*, and FcαRI colored in *salmon*. IgA1-Fc has a CH2-resident and a tailpiece *N*-glycan (shown in *lighter purple*; *N*-glycosylation sites depicted in *spheres* and *N*-glycans depicted as *sticks*). FcαRI has six potential *N*-glycosylation sites at Asn-44, Asn-58, Asn-120, Asn-156, Asn-165, and Asn-177 shown in *spheres*. The two *N*-glycosylation sites Asn-165 and Asn-177, which are hardly or not occupied, are colored in a *lighter color*. Complex (GnGn) *N*-glycans of IgA1-Fc and oligomannosidic (Man_9_) *N*-glycans of FcαRI are shown as *sticks. B*, five different tailpiece conformations (each shown in a different color) were aligned to the model where the backbone is shown as a cartoon, the tailpiece *N*-glycosylation site is marked in *spheres*, and glycans are shown as *sticks. C*, *N*-glycosylation sites Asn-165 and Asn-177 show the lowest SASA with ∼67 Å^2^, rendering the site inaccessible for *N*-glycosylation.

To obtain more insights into the role of *N*-glycosylation as well as the mechanisms that govern the IgA–FcαRI interaction, a molecular model was generated based on the IgA1-Fc–FcαRI crystal structure, the in-solution structure of the full-length IgA1 determined by small-angle X-ray scattering (SAXS), and addition of the IgA1 and FcαRI *N*-glycans. Therefore, iterative alignment of an *N*-glycan library was applied to the used *N*-glycosylation sites, and the obtained structures were minimized. One of the lowest-energetic conformations was selected (Fig. 7*A*). A possible explanation for the discrepancy in the binding stoichiometry as observed in the crystal structure (Protein Data Bank (PDB) code 1ow0) as well as ultracentrifugation studies (12, 30) and as determined in this work might be the absence of the IgA1's tailpiece in previous binding stoichiometry determinations (12, 13, 30, 34). Solution structures based on SAXS data sets (provided by David W. Wright) (36) suggested five alternative tailpiece conformations (Fig. 7*B*). These different tailpiece conformations of IgA1 exist in a mixture, causing the complex formation to follow a conformational selection. Thus, FcαRI binding will only occur when the IgA1-Fc tailpiece has a suitable binding conformation resulting in a mixture of 1:1 and 1:2 complexes.

Furthermore, the model offers an explanation for the unoccupied site Asn-177 and the partially glycosylated site Asn-165 discovered by site-specific glycopeptide analysis of FcαRI. Therefore, an oligomannosidic (Man_9_ structure) motion library was used on the glycosylated regions, and the accessibility of the experimentally unoccupied sites was investigated (Fig. 7*C*). The interference of *N*-glycans at sites Asn-44 and Asn-156 with Asn-177 and in a similar way the interference of the *N*-glycan at Asn-120 with Asn-165 make them likely inaccessible for the oligosaccharyltransferase complex catalyzing the *N*-glycosylation reaction. The solvent-accessible surface area (SASA) is ∼67 Å^2^ for Asn-165 and Asn-177 and ranges from 90 to 120 Å^2^ for the rest of the *N*-glycosylation sites. Because protein *N*-glycosylation occurs mainly cotranslationally in higher eukaryotes (37), the transfer of *N*-glycans to preceding *N*-glycosylation sites may sterically hinder the transfer of other *N*-glycans or modulate the folding of the receptor to reduce the SASA at specific sites.

## Discussion

The detailed biophysical and biochemical characterization of the different IgA glycoforms showed that modification of the *N*-glycans does not affect the overall structure and integrity but the thermal stability of the protein. This is in accordance with our previous study where we showed that plant- and HEK-derived IgA variants exhibit differences in thermal stability despite having identical amino acid sequences (18). The observed shifts of the transition midpoint temperatures of the CH2 domain of different IgA glycoforms produced in plants as well as different glycoforms produced in HEK293F cells suggest that complex *N*-glycans are beneficial for the thermal stability of IgA. Besides the various *N*-glycans found on IgA, IgA1 additionally has nine potential *O*-glycosylation sites in its extended proline-rich hinge region of which six are found to be occupied with mucin-type *O*-glycans in HEK293F-produced IgA1. On the hinge region of plant-produced IgA1, in contrast, conversion of proline residues to hydroxyproline and the presence of additional pentoses are observed (18, 25, 26). Thus, the destabilization of the CH2 domain of the different plant-derived compared with the HEK293F-produced IgA1 glycoforms could not only result from different *N*-glycan composition but also from the plant-specific hinge region modifications.

The crystal structure of the Fc domain of IgA1 in complex with two FcαRI suggests not only a role of the IgA *N*-glycans for structural properties of the protein but also a possible direct interaction with the FcαRI (12). Although the Fc domain of IgA generally resembles the Fc of IgG, the position of the CH2 domain *N*-glycan differs dramatically. In contrast to the IgG *N-*glycan, which is found between the upper Fc domains, the CH2-resident IgA *N*-glycan is located on the external surface and approaches within 8 Å of the FcαRI in the crystal structure (12). However, the complete *N*-glycan is not resolved, and thus the *N*-glycan could directly contact FcαRI. Previous studies reported contradictory results on the importance of the CH2-resident *N*-glycan (13–17). With our set of IgA variants with tailored *N*-glycans, we conducted quantitative SPR analysis to solve this controversy. These experiments showed that different glycoforms of all IgA isotypes do not significantly influence FcαRI binding. Apart from FcαRI, it has been proposed that IgA binds to multiple cellular receptors (38). Although terminal sialylation likely reduces the binding to asialoglycoprotein receptors and thus slows down the fast clearance of IgA (39), the contribution of *N*-glycans to the interaction with other receptors remains to be shown.

The role of glycosylation in antibody–receptor interaction has been traditionally focused on the function of the *N*-glycans of the antibody rather than the receptor. However, recent studies have led to renewed interests and focused on the role of the Fcα receptor *N*-glycans in IgG interaction and subsequent immune responses (10). As seen in the molecular model, the elongated complex-type human *N*-glycan is right at the interface and could directly influence the interaction of IgA with the Fcα receptor. Very little information exists as to the exact nature of the *N*-glycans found on FcαRI in humans. This is attributable to the limited possibilities to access sufficient quantity of the native receptor from neutrophils, monocytes, or eosinophils and represents a significant barrier for structure–function studies. Due to glycosylation with several potential *N*- and *O*-glycans, the mass of the 32-kDa protein can range from 55 to 100 kDa in cells of myeloid lineage. These differences suggest extensive and cell type–specific glycosylations (40–42) that have also been reported for other Fc receptors (10).

Site-specific *N*-glycan analysis of the recombinant FcαRI revealed that not all six *N*-glycosylation sites are fully occupied. The underglycosylation of the C-terminal *N*-glycosylation sites might be explained by restricted accessibility of the oligosaccharyltransferase complex due to protein folding or low solvent-accessible surface area as seen in our molecular model. However, the *N*-glycosylation site Asn-58 that is closest to the IgA interaction surface is fully occupied and exhibits branched complex *N*-glycans with or without a bisecting GlcNAc, sialic acid, and fucose on the nonreducing end. Mutation of Asn-58 increases binding of IgA to FcαRI, and a similar effect is observed for desialylated FcαRI (10, 43). Consistent with these findings, the desialylated recombinant FcαRI bound slightly better to all tested IgA glycoforms. The effect of desialylation on binding was more pronounced in previous reports where the receptor was produced in CHO cells. In addition to more efficient sialylation, CHO cells attach sialic acid in α2,3-linkage instead of the α2,6-linked sialic acid that is the predominant form in HEK293F. A 3-fold increase in binding affinity could be obtained by modifying the receptor glycans toward oligomannosidic *N*-glycans. Of all tested glycoforms, the receptor variant having single GlcNAc residues bound best with a 10-fold increase of affinity. This is interesting because CD spectroscopy revealed structural alterations in this variant, whereas desialylation or oligomannosidic *N*-glycans did not affect the overall structural conformation or thermal stability of the receptor. However, complex formation did not only stabilize the CH2 domain of IgA1 but also compensated for the loss of stability in the FcαRI_GlcNAc_ variant. This is in accordance with previously reported loss of IgA-Fc intra- and interdomain flexibility upon binding of the FcαRI, which might cause higher melting temperatures (34). Supported by our molecular model of the IgA1–FcαRI complex that includes all *N*-glycosylation sites, we propose that differential binding of the receptor glycoforms could either result from changes in surface charge by steric hindrance or, in the case of the FcαRI_GlcNAc_ variant, also from conformational changes. Thus, the *N*-glycosylation state of the receptor may not be essential for the formation of the ligand-binding site *per se*, but it affects the affinity to the ligand. This is particularly interesting because different cell types seem to glycosylate the receptor in a cell type–specific manner (40–42) and thus might modulate the potency of receptor signaling.

An unexpected finding is the discrepancy of the stoichiometry of full-length monomeric IgA variants in complex with FcαRI compared with previous studies. Cocrystallization of an IgA1-Fc construct lacking the tailpiece with FcαRI identified a clear 1:2 binding stoichiometry (12). Further ultracentrifugation studies and SPR experiments confirmed this observation (13, 30). Here, SPR and SEC-HPLC-MALS measurements revealed a 1:1 stoichiometry. For SEC-HPLC-MALS, IgAs were mixed with FcαRI in a 1:4 molar ratio with an adequately high concentration that exceeds the low-affinity *K_D_* of the second binding event postulated in previous studies more than 10-fold. This concentration theoretically should allow the occupation of both identical FcαRI-binding sites of IgA. A deletion of IgA lacking the Fab domain led to the same conclusion. SEC is a separation technology where the two interaction partners migrate differently after dissociation. By contrast, during ITC analysis, the binding partners are in equilibrium in a closed reaction chamber similar to the conditions during crystallization processes, and dilution and separation effects found in SEC experiments are not present. Therefore, the high concentrations of the interaction partners should allow binding to a low-affinity site as well. ITC results clearly showed a 1:1.3 binding stoichiometry with a single transition, suggesting the presence of a mixture of 1:1 and 1:2 complexes, with both binding events of FcαRI being very similar. A likely explanation for the discrepancy might be the absence of the IgA1's tailpiece in the protein used for crystallization and ultracentrifugation studies. Previously, differences in binding affinities of the receptor to monomeric IgA1 from serum and recombinant IgA1-Fc lacking the tailpiece and the hinge region could be observed in SPR experiments when a 1:2 model was applied (13). Because the hinge region is 20–30 Å away from the interaction surface, it was hypothesized that the tailpiece plays a role in the differences in affinity (13). Based on our observation in ITC experiments and our molecular model that was obtained by superposition of the complex crystal structure and SAXS data of full-length IgA, we propose that the tailpiece does not necessarily change the interaction surface but that it exists in different conformations, allowing binding of a second FcαRI only if a suitable condition is met. Understanding the mode of IgA–FcαRI binding is important as a higher stoichiometry has recently been linked to enhanced immunoreceptor tyrosine-based activating motif (ITAM) signaling that led to potent neutrophil effector functions (44). A predominant 1:1 binding would therefore suggest that the postulated avidity effects are not necessarily responsible for the superiority of IgA-elicited tumor killing by neutrophils compared with poor IgG-mediated killing.

## Experimental procedures

### Construct design and cloning

All constructs used for the expression of anti-HER2-binding IgA1, IgA2m(1), and IgA2m(2) isotypes in *N. benthamiana* and HEK293 cells have been described in detail recently (18). The vectors for the transient expression of the different anti-HER2 IgA isotypes in HEK293F cells were constructed by flanking the previously described codon-optimized DNA sequences of the heavy chains (α-HC) and the κ light chain (κ-LC) with DNA sequences encoding the signal peptides MELGLSWIFLLAILKGVQC and MDMRVPAQLLGLLLLWLSGARC, respectively, and the restriction sites BamHI and SalI. The synthesized DNA was then amplified by PCR with the primers gWiz_1F (TCTGAGCAGTACTCGTTGCTG)/gWiz_1R (AACAACAGATGGCTGGCAAC). The corresponding coding regions for the heavy chains and the κ light chain were then separately cloned into the BamHI/SalI sites of the mammalian expression vector gWIZ (Genlantis, San Diego, CA). The codon-optimized sequence for the expression of the Fc domain of IgA1 in HEK293F, including the signal peptide of the α-HC and BamHI/SalI restriction sites, was synthesized by GeneArt (Thermo Fisher Scientific) and cloned into the gWIZ vector as described above.

The codon-optimized DNA sequence for the expression of the extracellular domain of the human FcαRI (P24071.1) with a C-terminal penta-His tag and the same N-terminal signal peptide for secretion of the κ-LC in HEK293F was synthesized by GeneArt. The sequence was amplified with primers String_10F (CTTCCGGCTCGTTTGGTCGAC)/String_2R (AAAACCCTGGCGGGATCC), digested with SalI/BamHI, and cloned into SalI/BamHI-digested gWIZ vector.

The sequence coding for the catalytic domain of human GnTII was amplified by PCR from the vector pPT2M-GnTII with primers Hs-GnTII-9F (GGATCCGAGGCGGACAACCTGACGCTGCG)/Hs-GnTII-10R (CTCGAGTCACTGCAGTCTTCTATAACTTTTAC) (26). The PCR product was digested with BamHI/XhoI and cloned into the BamHI/SalI-digested p20-RST-CTS-Fc vector, thereby removing the Fc-GFP insert and generating pPT2-RST-HsGnTII vector containing the CTS region from rat α2,6-sialyltransferase (RST) for *trans*-Golgi targeting (45).

The sequence and cloning of *N. benthamiana* β-hexosaminidases (NbHEXOs) has been described previously (46). To obtain constructs expressing the catalytic domain of NbHEXO3 with an N-terminal *N. benthamiana* chitinase signal peptide (23), the NbHEXO3 sequence was amplified from the p31-NbHEXO3 vector using primers Nb-Hexo3-F7 (TATAGGATCCAAGTACCCTGATACCTCTGGAATT)/Nb-Hexo3-R7 (TATAAGATCTTTGCTGATAGCAAGAACCTGGATC). The PCR product was digested with BamHI/BglII and cloned into the BamHI-digested p31 vector to obtain p31-NbHEXO3(CD). The signal peptide was obtained by PCR amplification from *N. benthamiana* cDNA with primers Nb-Chi_F1 (TATATCTAGAATGAGGCTTAGAGAATTCACAG)/Nb-Chi_R1 (TATAGGATCCTGCCGAGGCAGAGAGTAGGAGAGA), digestion of the PCR product with XbaI/BamHI, and cloning into XbaI/BamHI-digested p31-NbHEXO3(CD) to generate p31-SP-NbHEXO3(CD) for targeting of NbHEXO3 to the apoplast. A construct with catalytic domain 2 of NbHEXO3 fused to the RST CTS region was obtained by amplification of p31-NbHEXO3 with primers Nb-Hexo3-F9 (TATAGGATCCTTGAAGATATGGCCGATGCCACTA)/Nb-Hexo3-R7 (TATAAGATCTTTGCTGATAGCAAGAACCTGGATC), digestion with BamHI/BglII, and cloning into BamHI-digested p31 to obtain p31-NbHEXO3(CD2). The RST CTS sequence was excised from p20-RST-CST-Fc with XbaI/BamHI and cloned into XbaI/BamHI sites of p31-NbHEXO3(CD2) to generate p31-RST-NbHEXO3(CD2) for targeting to the *trans*-Golgi. Binary vectors for the expression of proteins involved in galactosylation, CMP-sialic acid biosynthesis, Golgi transport, and sialic acid transfer were available from previous studies (20).

### Expression and purification of IgA glycoforms

For the expression of different recombinant IgA glycoforms in 5–6-week-old *N. benthamiana* ΔXT/FT plants, syringe-mediated agroinfiltration was used (18, 21). The recombinant IgAs were either expressed alone or coinfiltrated with the vectors coding for the respective proteins for *N*-glycan modifications. Thereby, an OD_600_ of 0.15 was used for the κ-LC, α-HC, and GnTII. All remaining constructs involved in glycan modification were added with a final OD_600_ of 0.05. After 4 days, infiltrated leaf material was harvested, and the clarified crude extract was prepared for IgA purification as described before (18).

For the transient expression of the different IgA isotypes in HEK293F cells, cultures were maintained and transfected according to the manufacturer's manual in FreeStyle^TM^ expression medium (Thermo Fisher Scientific). High-quality plasmid preparations were obtained with the PureYield^TM^ Plasmid Midiprep System (Promega). For the transfection of a 200-ml culture with a cell density of 1.0 × 10^6^ cells/ml, a total of 200 μg of plasmid DNA, consisting of 100 μg of κ-LC and 100 μg of the respective α-HC, were mixed in 4 ml of OptiPro^TM^ SFM medium (Thermo Fisher Scientific). Another 4 ml of OptiPro SFM medium containing 2.5 μg/ml linear polyethylenimine (PEI) (Polysciences Inc., Germany) were added to the DNA solution and incubated for 15 min before the mixture was slowly titrated to the cell culture. To obtain IgA isotypes with oligomannosidic *N*-glycans, the class I α-mannosidase inhibitor kifunensine (Santa Cruz Biotechnology) was added to the cell culture in a final concentration of 10 μm. The cultures were incubated for 7 days at 37 °C in a humidified atmosphere with 8% CO_2_ on an orbital shaker rotating at 135 rpm. The supernatant containing the secreted soluble protein was harvested by centrifugation at 25,000 × *g* for 30 min at 4 °C and additionally filtrated through a 0.45-μm Durapore membrane filter (Merck Millipore, Germany). IgA from clarified *N. benthamiana* ΔXT/FT leaf extract and supernatant of HEK293F cells was purified with IgA CaptureSelect affinity resin (Thermo Fisher Scientific) followed by an SEC step as described (18).

### Expression and purification of FcαRI glycoforms

For the recombinant production of different glycoforms of the extracellular domain of the FcαRI, HEK293F cells were cultured and transfected as described in the previous section. To obtain FcαRI with oligomannosidic *N*-glycans (FcαRI_Man9_), the cultures were transfected in the presence of 10 μm kifunensine. The cell culture supernatants were harvested after 6 days, prepared for purification as described above, and diluted 1:2 in loading buffer (20 mm Tris, 500 mm NaCl, and 10 mm imidazole). The solution was loaded onto a 5-ml HisTrap HP column (GE Healthcare) equilibrated with 5 column volumes of loading buffer, and bound protein was eluted by applying 250 mm imidazole. Eluted fractions containing the protein of interest were pooled and dialyzed overnight against Dulbecco's phosphate-buffered saline (PBS) (Sigma-Aldrich) supplemented with 200 mm NaCl at 4 °C using SnakeSkin dialysis tubing with a 10-kDa molecular mass cutoff (Thermo Fisher Scientific). Protein samples were then further concentrated using a 10-kDa Amicon Ultra centrifugal filter (Merck Millipore). To obtain FcαRI_desia_, 100 μg of FcαRI were digested with 1000 units of neuraminidase (New England Biolabs) according to the manufacturer's protocol. To generate a variant of FcαRI with a single GlcNAc at each *N*-glycosylation site (FcαRI_GlcNAc_), 500 μg of FcαRI_Man9_ were digested with 4000 units of endoglycosidase H (New England Biolabs) followed by endoglycosidase H removal with amylose resin (New England Biolabs). The different FcαRI glycoforms were then subjected to SEC on a HiLoad 16/600 Superdex 200 prep grade column (GE Healthcare) equilibrated with the same buffer used for dialysis.

### SDS-PAGE

For reducing or nonreducing SDS-PAGE, a total of 5 μg of purified protein was either loaded on a 4–15% Mini-PROTEAN® TGX^TM^ gel (Bio-Rad) or a 10% polyacrylamide gel. Separated proteins were detected by Coomassie Brilliant Blue staining.

### SE-HPLC-MALS

To verify the conformational integrity and molecular weight of purified IgAs, FcαRI, and IgA–FcαRI complexes, HPLC coupled to a size-exclusion chromatography column (Superdex 200 10/300 GL column, GE Healthcare) combined with multiangle light scattering was carried out as described previously (18). HPLC (Shimadzu Prominence LC20) was equipped with MALS (WYATT Heleos Dawn8+ QELS; software ASTRA6), a refractive index detector (RID-10A, Shimadzu), and a diode array detector (SPD-M20A, Shimadzu). Single protein measurements were performed by injection of a total amount of 25 μg of protein. For the determination of the mass of IgA–FcαRI complexes, the different IgA variants were mixed with the receptor in a molar ratio of 1:4 starting from 25 μg of the respective IgA.

### DSC

The thermal stability of the IgA variants, FcαRI, and different IgA–FcαRI complexes was analyzed by DSC using a MicroCal PEAQ-DSC (Malvern, UK). SEC-purified IgA samples were diluted to a concentration of 5 μm, FcαRI was diluted to 10 μm, and IgA–FcαRI complexes were mixed in a 1:1 ratio to form a complex with a 10 μm concentration in PBS buffer. Samples were filtered through a 0.1-μm Ultrafree-MC filter (Merck Millipore) before measurements in the temperature range of 30–100 °C with a heating rate of 1 °C/min were carried out. Buffer baselines were subtracted, normalized for protein concentration, and fitted with a non-two-state thermal unfolding model using the MicroCal PEAQ-DSC software.

### CD spectroscopy

CD spectroscopy was performed using a Chirascan^TM^ CD spectrometer (Applied Photophysics, UK). The instrument was flushed with a nitrogen flow of 5 liters/min. The different FcαRI glycoforms were brought to an absorbance at 280 nm of 0.8 in 5 mm phosphate buffer, pH 7.4. Samples were measured in a cuvette with a path length of 1 mm in the far-UV region ranging from 190 to 260 nm, a 5-nm/s scan speed, and a 3-nm bandwidth.

### N- and O-glycan analysis

A total of 20 μg of purified protein was reduced, *S*-alkylated, and digested with trypsin (Promega). If required, samples were additionally digested with the endoprotease Asp-N (Sigma-Aldrich). Glycopeptides were then analyzed by capillary reversed-phase chromatography and electron-spray MS using a Bruker Maxis 4G Q-TOF instrument as described previously (18). Site-specific glycosylation occupancy was calculated using the ratio of deamidated to unmodified peptide determined upon *N*-glycan release with peptide:*N*-glycosidase A (Europa Bioproducts).

### SPR spectroscopy

Binding experiments of IgA glycoforms to different FcαRI variants were performed using a Biacore T200 (GE Healthcare). All measurements were conducted with a Protein L sensor chip (GE Healthcare), and all sample dilutions were prepared in 1× PBS, 0.05% Tween, and 0.1% BSA. Capture of the different IgA variants on the Protein L surface was performed for 60 s with a concentration of 2 μg/ml and a flow rate of 10 μl/s. Flow cell 2 remained unmodified to serve as a reference cell for the subtraction of systematic instrument noise and drift. FcαRI binding curves were generated in single-cycle kinetic experiments at five different concentrations ranging from 31.5 to 500 nm with 60-s association and 60-s dissociation time at a flow rate of 10 μl/min. After each run, surface regeneration was accomplished using 10 mm glycine, pH 1.7, for 120 s at a flow rate of 30 μl/min. Binding affinities (*K_D_*) were calculated with Biacore T2 Evaluation software using a 1:1 binding model. All experiments were repeated as four independent kinetic runs from two different IgA and FcαRI preparations.

### ITC

ITC measurements were performed on a MicroCal Automated PEAQ-ITC (Malvern Instruments, UK) to investigate the binding stoichiometry of IgA–FcαRI complexes in solution. All samples were prepared in PBS buffer, pH 7.4; centrifuged at 20,000 × *g* for 10 min at room temperature; and filtered through a 0.1-μm Ultrafree-MC filter prior to measurement. The sample cell was filled with 10 μm IgA solution and titrated with a 160 μm stock solution of the respective FcαRI. Titrations were conducted at 25 °C using an initial injection of 0.1 μl followed by 19 successive injections of 1.5 μl with a 150-s injection interval. Binding stoichiometry (*N*) was determined using the MicroCal PEAQ-ITC analysis software.

### Molecular modeling and simulation

The molecular model of the IgA1-Fc in complex with FcαRI was made using the complex crystal structure with PDB code 1ow0 (12). The model of IgA1-Fc, including the CH2-resident and tailpiece *N*-glycosylation sites, was based on data from SAXS studies obtained upon request (36). The variable regions of IgA1 were modeled with the PIGSPro web server, which is a predictive modeling tool specialized for Igs (47). Complex (GnGn-type) and oligomannosidic (Man_9_GlcNAc_2_) *N*-glycans were attached to IgA1 and FcαRI, respectively. For the generation of the 3D models of the glycan structures, an enhanced sampling method, consisting of two steps, was used. First, the biased potentials for the glycosidic linkages of disaccharides are built up followed by a sampling step to cover the conformational space of the larger glycans (48, 49). This approach enables the creation of motion libraries of GnGn-type and Man_9_GlcNAc_2_ glycans. An example of the latter is shown in Fig. S8. After stepwise alignment of glycan motion libraries at each *N*-glycosylation site, a short minimization with conjugate gradient (50) using the GROMOS11 biomolecular simulation package (http://www.gromos.net)^3^ was applied (51). Molecular interactions were described according to the 53A6glyc parameter set (52, 53) of the GROMOS force field for carbohydrates and the 54A8 parameter set (54) for the protein. After each alignment of the glycan, the conformation with the lowest energy was selected. The SASAs for the *N*-glycosylation sites were calculated using the PDBePISA web server (55).

## Author contributions

K. G., A. T., J. N., C. Oostenbrink, C. Obinger, and R. S. conceptualization; K. G. data curation; K. G., A. T., D. M., and R. S. formal analysis; K. G., A. T., and R. S. investigation; K. G. and A. T. visualization; K. G., A. T., D. M., F. A., C. Oostenbrink, C. Obinger, and R. S. methodology; K. G. and R. S. writing-original draft; K. G., A. T., J. N., F. A., C. Oostenbrink, C. Obinger, and R. S. writing-review and editing; J. N., F. A., C. Oostenbrink, C. Obinger, and R. S. supervision; C. Obinger and R. S. funding acquisition; R. S. resources; R. S. project administration.

## Supplementary Material

Supporting Information

## Footnotes

3 Please note that the JBC is not responsible for the long-term archiving and maintenance of this site or any other third party hosted site.

## Footnotes

2 The abbreviations used are:

FcαRI: Fcα receptor
HER2: human epidermal growth factor receptor 2
SE-HPLC-MALS: size-exclusion chromatography coupled to multiangle light scattering
DSC: differential scanning calorimetry
ITC: isothermal titration calorimetry
SPR: surface plasmon resonance
GnGn: GlcNAc_2_Man_3_GlcNAc_2_
GnTII: *N*-acetylglucosaminyltransferase II
MM: Man_3_GlcNAc_2_
AA: terminally galactosylated
NaNa: sialylated
HEK: human embryonic kidney
desia: desialylated
SAXS: small-angle X-ray scattering
SASA: solvent-accessible surface area
CHO: Chinese hamster ovary
HC: heavy chain
LC: light chain
CTS: cytoplasmic, transmembrane, and stem
RST: rat α2,6-sialyltransferase
NbHEXO: *N. benthamiana* β-hexosaminidase
SFM: serum-free medium
SEC: size-exclusion chromatography.
