# Supplementary material for: Distinct Fcα receptor N-glycans modulate the binding affinity to immunoglobulin A (IgA) antibodies
Source: J Biol Chem. 2019 Jul 30;294(38):13995–4008. doi: 10.1074/jbc.RA119.009954 (PMC6755811; doi:10.1074/jbc.RA119.009954)
Supplement: Supporting Information [file supp_294_38_13995__index.html]

Distinct Fc alpha receptor N-glycans modulate the binding affinity to immunoglobulin A (IgA) antibodies — Role of IgA and FcαRI N-glycans — Distinct Fcα receptor N-glycans modulate the binding affinity to immunoglobulin A (IgA) antibodies — Role of IgA and FcαRI N-glycans — Supporting Information 

# Distinct Fcα receptor *N*-glycans modulate the binding affinity to immunoglobulin A (IgA) antibodies

## Supporting Information

- Supporting Information (to be published online) - Supplementary Figures 1-8 and Tables 1-2
