# Supplementary material for: Distinct Fcα receptor N-glycans modulate the binding affinity to immunoglobulin A (IgA) antibodies
Source: J Biol Chem. 2019 Jul 30;294(38):13995–4008. doi: 10.1074/jbc.RA119.009954 (PMC6755811; doi:10.1074/jbc.RA119.009954)
Supplement: Supporting Information [file supp_RA119.009954_153940_1_supp_367194_pv3bpy.pdf]

SUPPORTING INFORMATION

Distinct Fc alpha receptor N-glycans modulate the binding affinity to immunoglobulin A (IgA) antibodies

Kathrin Göritzer[1], Aysegül Turupcu[2], Daniel Maresch[3], Jan Novak[4], Friedrich Altmann[3], Chris Oostenbrink[2], Christian Obinger[3], and Richard Strasser[1]*

From the [1]Department of Applied Genetics and Cell Biology, University of Natural Resources and Life Sciences, Muthgasse 18, A-1190 Vienna; [2]Department of Material Sciences and Process Engineering, University of Natural Resources and Life Sciences, Muthgasse 18, A-1190 Vienna; [3]Department of Chemistry, Division of Biochemistry, University of Natural Resources and Life Sciences, Muthgasse 18, A-1190 Vienna; [4]Department of Microbiology, University of Alabama at Birmingham, 845 19th Street, Birmingham, AL 35294

Running Title: *Role of IgA and FcαRI N-glycans*

*To whom correspondence should be addressed: Richard Strasser, Department of Applied Genetics and Cell Biology, University of Natural Resources and Life Sciences, Muthgasse 18, A-1190 Vienna; richard.strasser@boku.ac.at; Tel. +43-1-47654-94145; Fax. +43-1-47654-94009

Keywords: antibody, Fc receptor, glycobiology, glycoprotein structure, glycosylation, immunoglobulin A, molecular dynamics, recombinant protein expression, adaptive immunity, posttranslational modification

---

Figure S1
Figure S2
Figure S3
Figure S4
Figure S5
Figure S6
Figure S7
Figure S8
Table S1
Table S2

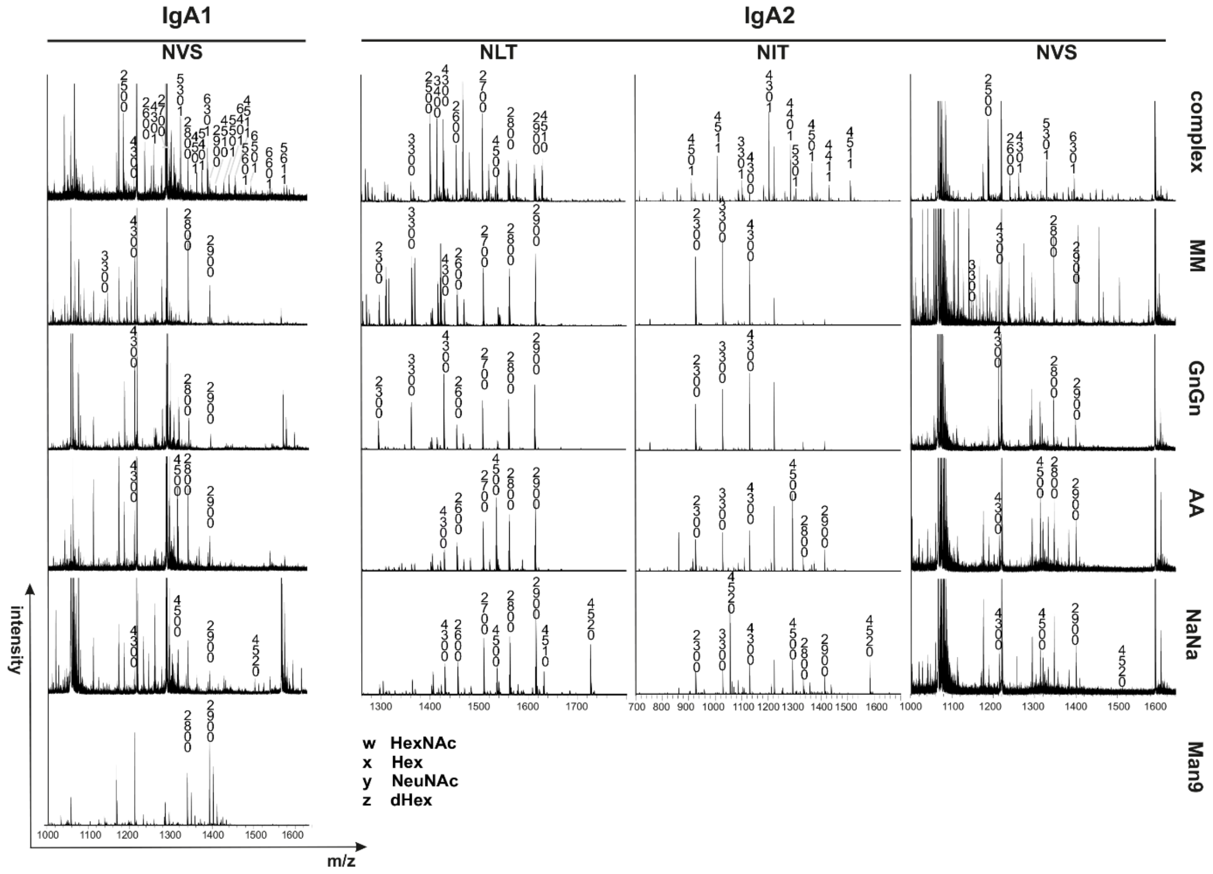

Figure S1. MS-analysis of different IgA1 and IgA2 glycoforms. Representative MS-spectra ($[M+3H]^{3+}$) of the tryptic glycopeptides “LAGKPTHVNVSVVMAEVDGTCY” of IgA1 as well as “LSLHRPALEDLLLGSEANLTCTLTGLR”, “TPLTANITK” and “LAGKPTHVNVSVVMAEVDGTCY” of IgA2m(2) containing the Fc-glycosylation sites derived from the alpha chain of the different purified IgA1 and IgA2m(2) glycoforms. MS-spectra of IgA2m(1) are not shown, but resemble very accurately MS-spectra of IgA2m(2).

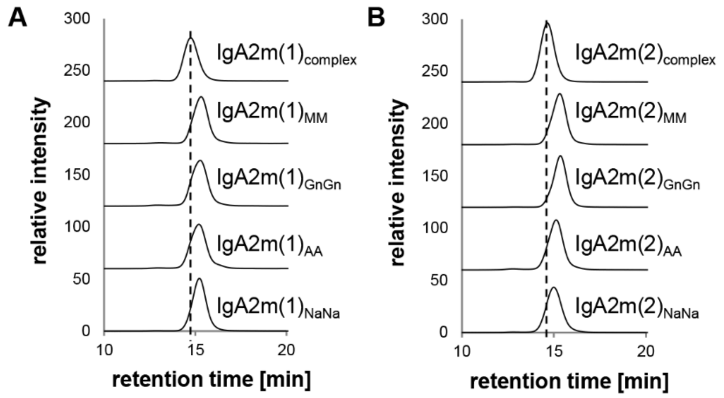

Figure S2. SE-HPLC measurements of the different IgA2m(1) (A) and IgA2m(2) (B) glycoforms. In order to facilitate comparison between the different variants the elution time of IgA2m(1)$_{complex}$ and IgA2m(2)$_{complex}$ are marked with dashed lines.

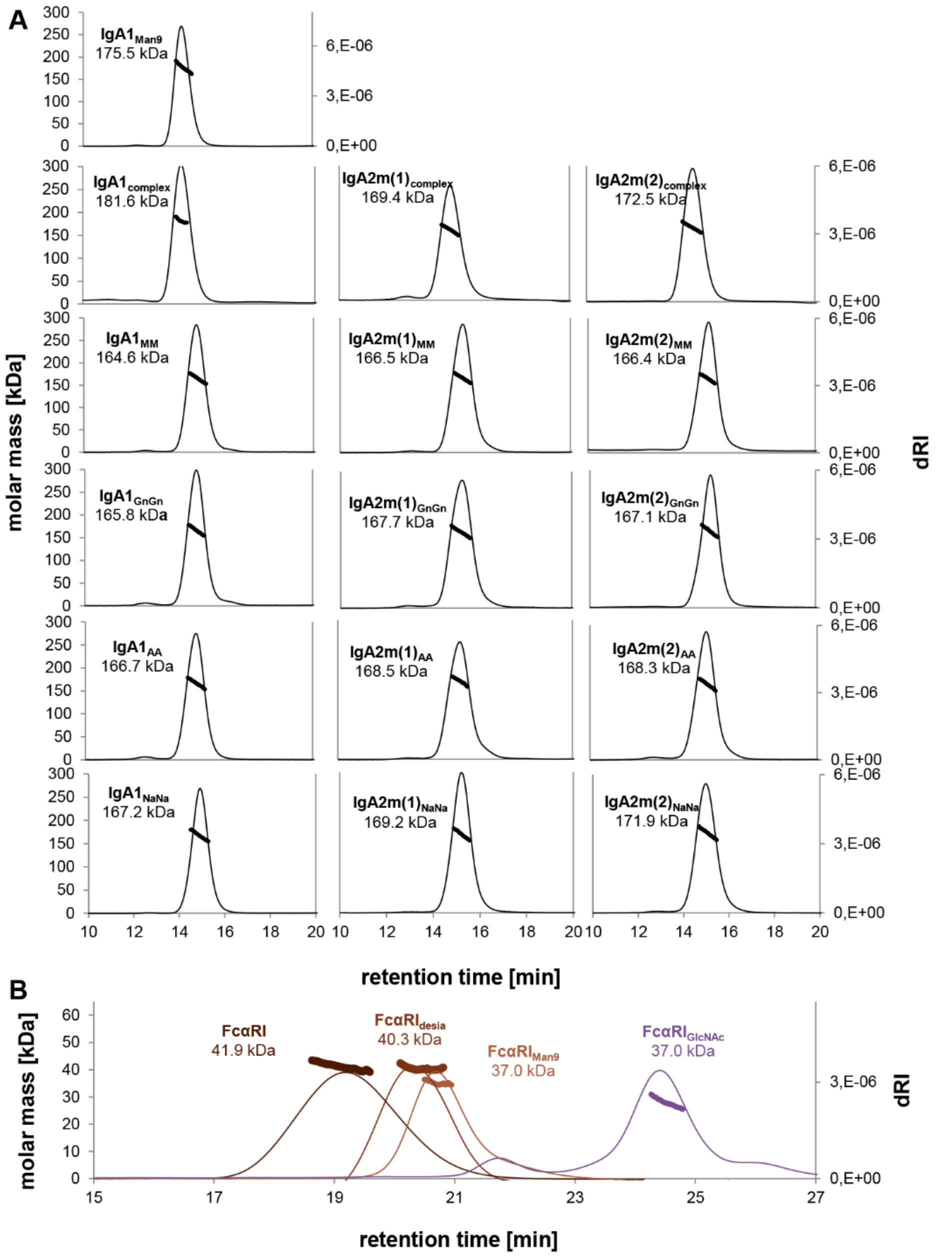

Figure S3. Representative SE-HPLC-MALS measurements of each IgA (A) and FcαRI (B) variant to determine the molar mass in solution.

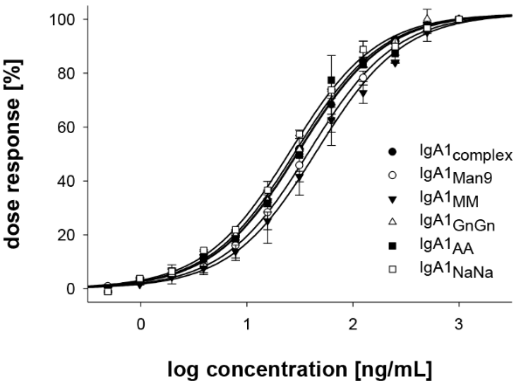

	$IgA1_{complex}$	$IgA1_{Man9}$	$IgA1_{MM}$	$IgA1_{GnGn}$	$IgA1_{AA}$	$IgA1_{NaNa}$
**EC_{50} [ng/mL]**	30.9	38.9	45.7	28.8	30.2	25.11

Figure S4. Binding of different IgA1 glycoforms to HER2. Determination of EC_{50} values of IgA1glycoforms was determined by ELISA as reported in a previous study [18] and were calculated as global fit of three repetitions.

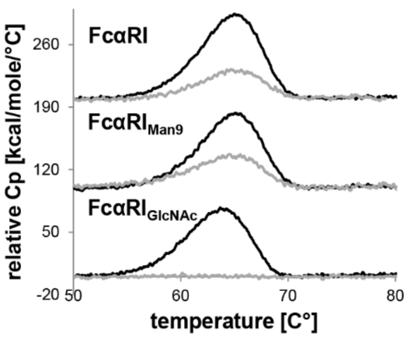

Figure S5. DSC (re-)scans of different FcαRI glycoforms. Bold lines show the raw date of DSC thermograms, whereas the thin lines are the rescan of each measurement.

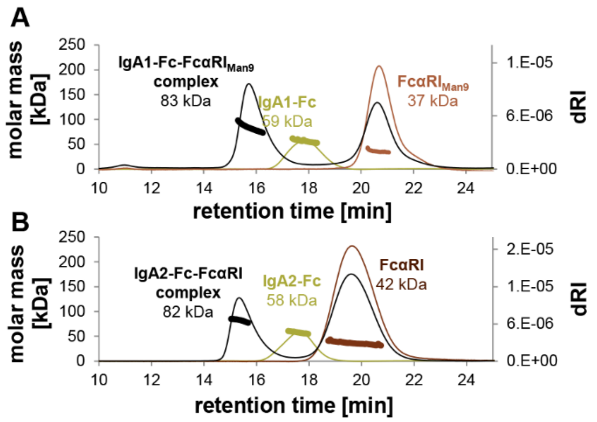

Figure S6. SE-HPLC-MALS to determine the mass of IgA-Fc-FcαRI complexes. (A) Overlay of the elution profile of IgA1-Fc (green), FcαRI$_{Man9}$ (light brown) and a mixture of IgA1-Fc and FcαRI$_{Man9}$ in a ratio of 1:4 (black). (B) Overlay of the elution profile of IgA2-Fc (green), FcαRI (brown) and a mixture of IgA1-Fc and FcαRI in a ratio of 1:4 (black). Note that the depicted molar masses are derived from MALS measurements and thus do not exactly match the exact molar masses.

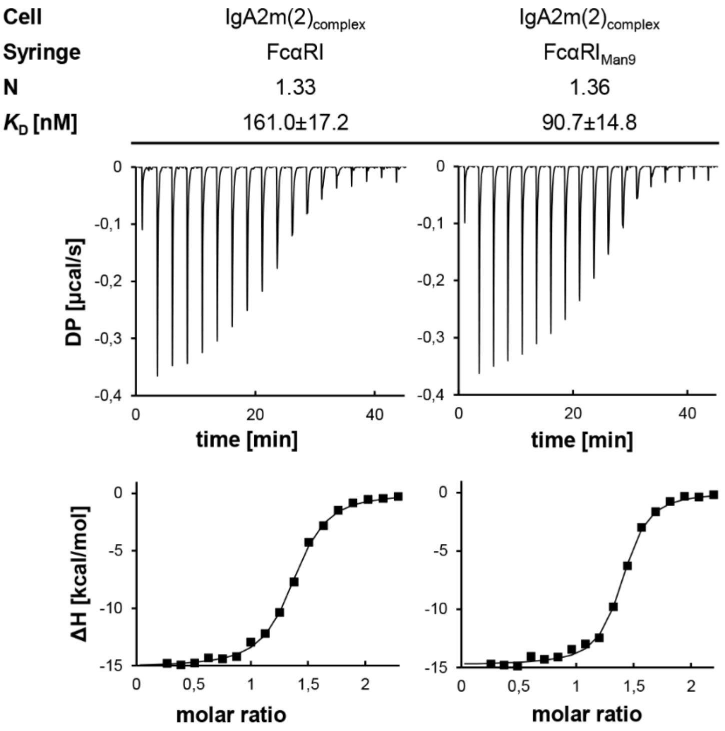

Figure S7. ITC measurements of IgA2m(2)$_{complex}$ with FcαRI and FcαRI$_{Man9}$. The upper panels show the raw data representing the response to 19 injections at 25°C and the lower panels the integrated data.

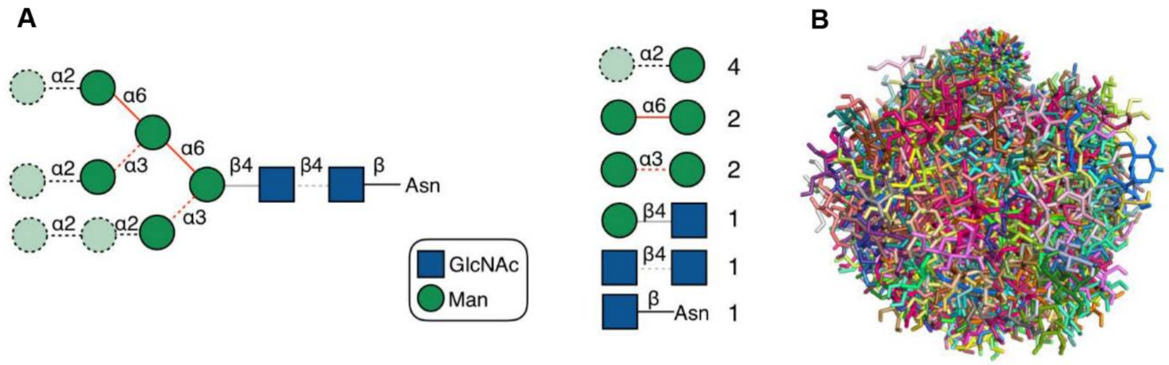

Figure S8. Generation of M9 distinct rotamer library. Using previously built local elevation potentials for each linkage, local elevation with umbrella sampling simulations (LEUS) were performed. For the M9 oligomannosidic (total 10+N linkages) N-glycan 6 different potentials were used; α-D-Man-(1→2)- α-D-Man (at 4 linkages); α-D-Man-(1→6)- α-D-Man (at 2 linkages); α-D-Man-(1→3)- α-D-Man(at 2 linkages); α-D-Man-(1→4)-β-D-GlcNAc (at 1 linkage); β-D-GlcNAc-(1→4)-β-D-GlcNAc (at 1 linkage) and β-D-GlcNAc→N (at 1 linkage). The adaptiveness of the LEUS method allows the use of already built potentials to different locations, which have the same linkage composition. (A) Composition of the M9 N-glycan is drawn with emphasized distinct linkages. On the right, distinct disaccharide linkages which were used in the generation of the local elevation potentials are depicted with number of their occurrence in the M9 glycan used in this study. (B) M9 high-mannose motion library is represented by different colors for each conformation (only 300 snapshots out of 1 million are shown here).

Table S1. Transition midpoint temperatures of the CH2 (T_m1), Fab (T_m2) and CH3 (T_m3) domains of IgA1, IgA2m(1) and IgA2m(2) glycoforms obtained from differential scanning calorimetry (DSC).

	T_m1 [°C]			T_m2 [°C]			T_m3 [°C]		
**IgA1$_{complex}$**	71.51	±	0.06	74.15	±	0.04	76.31	±	0.05
**IgA1$_{Man}$**	70.82	±	0.16	73.64	±	0.13	76.00	±	0.09
**IgA1$_{MM}$**	68.38	±	0.00	72.98	±	0.01	75.43	±	0.02
**IgA1$_{GnGn}$**	68.83	±	0.16	73.10	±	0.06	75.55	±	0.05
**IgA1$_{AA}$**	69.27	±	0.25	73.26	±	0.07	75.67	±	0.04
**IgA1$_{NaNa}$**	69.48	±	0.10	73.33	±	0.02	75.66	±	0.03
**IgA2m(1)$_{complex}$**	67.91	±	0.16	71.95	±	0.02	78.28	±	0.07
**IgA2m(1)$_{MM}$**	63.50	±	0.12	70.63	±	0.00	78.44	±	0.02
**IgA2m(1)$_{GnGn}$**	63.72	±	0.17	70.58	±	0.01	78.36	±	0.02
**IgA2m(1)$_{AA}$**	64.60	±	0.09	70.68	±	0.00	78.46	±	0.01
**IgA2m(1)$_{NaNa}$**	64.37	±	0.12	70.73	±	0.00	78.65	±	0.02
**IgA2m(2)$_{complex}$**	73.30	±	0.11	76.30	±	0.06	78.90	±	0.05
**IgA2m(2)$_{MM}$**	69.90	±	0.01	74.00	±	0.07	77.70	±	0.04
**IgA2m(2)$_{GnGn}$**	70.00	±	0.11	74.20	±	0.05	78.00	±	0.04
**IgA2m(2)$_{AA}$**	71.60	±	0.20	74.70	±	0.08	77.90	±	0.06
**IgA2m(2)$_{NaNa}$**	71.90	±	0.16	75.30	±	0.07	78.30	±	0.06

Table S2. Kinetic parameters for selected FcαRI IgA pairs. Rate constants are an average of four independent SPR experiments at 5 different concentrations.

ligand	analyte	k_{on} [(M s)$^{-1}$]			k_{off} [s^{-1}]			K_D [nM]		
**$IgA1_{MM}$**	FcαRI	3.5 x 10^5	±	1.0 x 10^5	0.07	±	0.01	187.43	±	3.41
**$IgA1_{NaNa}$**	FcαRI	5.2. x 10^5	±	4.2 x 10^4	0.07	±	0.01	149.26	±	1.68
**$IgA2m(1)_{MM}$**	FcαRI	4.0 x 10^5	±	7.6 x 10^4	0.07	±	0.01	187.76	±	7.24
**$IgA2m(1)_{NaNa}$**	FcαRI	3.9 x 10^5	±	2.5 x 10^4	0.06	±	0.01	172.53	±	5.07
**$IgA2m(2)_{MM}$**	FcαRI	2.8 x 10^5	±	9.9 x 103	0.06	±	0.00	225.29	±	8.54
**$IgA2m(2)_{NaNa}$**	FcαRI	3.0 x 10^5	±	3.8 x 10^4	0.06	±	0.00	205.74	±	2.56
**$IgA1_{MM}$**	$Fc\alpha RI_{GlcNAc}$	1.9 x 10^6	±	2.7 x 10^5	0.05	±	0.00	25.24	±	0.97
**$IgA1_{NaNa}$**	$Fc\alpha RI_{GlcNAc}$	2.3 x 10^6	±	6.1 x 10^5	0.06	±	0.01	25.76	±	1.22
**$IgA2m(1)_{MM}$**	$Fc\alpha RI_{GlcNAc}$	1.6 x 10^6	±	2.2 x 10^5	0.04	±	0.00	26.31	±	0.95
**$IgA2m(1)_{NaNa}$**	$Fc\alpha RI_{GlcNAc}$	1.7 x 10^6	±	2.2 x 10^5	0.04	±	0.00	24.20	±	0.93
**$IgA2m(2)_{MM}$**	$Fc\alpha RI_{GlcNAc}$	1.2 x 10^6	±	7.9 x 10^4	0.03	±	0.00	26.20	±	0.26
**$IgA2m(2)_{NaNa}$**	$Fc\alpha RI_{GlcNAc}$	1.3 x 10^6	±	1.4 x 10^5	0.03	±	0.00	25.22	±	0.76
